# Mass spectrometry-based proteomics in basic and translational research of SARS-CoV-2 coronavirus and its emerging mutants

**DOI:** 10.1186/s12014-021-09325-x

**Published:** 2021-08-12

**Authors:** Yasmine Rais, Zhiqiang Fu, Andrei P. Drabovich

**Affiliations:** Division of Analytical and Environmental Toxicology, Department of Laboratory Medicine and Pathology, Faculty of Medicine and Dentistry, University of Alberta, Edmonton, AB Canada

**Keywords:** Betacoronavirus, COVID-19, Emerging mutations, Immunoprecipitation, Mass spectrometry, Proteomics, SARS-CoV-2, Serology diagnostics, Targeted proteomics

## Abstract

Molecular diagnostics of the coronavirus disease of 2019 (COVID-19) now mainly relies on the measurements of viral RNA by RT-PCR, or detection of anti-viral antibodies by immunoassays. In this review, we discussed the perspectives of mass spectrometry-based proteomics as an analytical technique to identify and quantify proteins of the severe acute respiratory syndrome coronavirus 2 (SARS-CoV-2), and to enable basic research and clinical studies on COVID-19. While RT-PCR and RNA sequencing are indisputably powerful techniques for the detection of SARS-CoV-2 and identification of the emerging mutations, proteomics may provide confirmatory diagnostic information and complimentary biological knowledge on protein abundance, post-translational modifications, protein–protein interactions, and the functional impact of the emerging mutations. Pending advances in sensitivity and throughput of mass spectrometry and liquid chromatography, shotgun and targeted proteomic assays may find their niche for the differential quantification of viral proteins in clinical and environmental samples. Targeted proteomic assays in combination with immunoaffinity enrichments also provide orthogonal tools to evaluate cross-reactivity of serology tests and facilitate development of tests with the nearly perfect diagnostic specificity, this enabling reliable testing of broader populations for the acquired immunity. The coronavirus pandemic of 2019–2021 is another reminder that the future global pandemics may be inevitable, but their impact could be mitigated with the novel tools and assays, such as mass spectrometry-based proteomics, to enable continuous monitoring of emerging viruses, and to facilitate rapid response to novel infectious diseases.

## Background

The coronavirus disease 2019 (COVID-19) pandemic hit the world in November 2019 and quickly spread to all continents and nearly all countries. The COVID-19 disease was caused by the severe acute respiratory syndrome coronavirus 2 (SARS-CoV-2) and has resulted in over 128 million confirmed cases and 2.8 million deaths worldwide as of March 2021. Prior to the SARS-CoV-2, the SARS-CoV-1 (year 2002) and MERS-CoV (year 2012) epidemics claimed together about 1,600 lives. Enormous efforts were devoted worldwide to limit the spread of SARS-CoV-2, develop rapid diagnostics, mitigate severe and deadly clinical complications, and produce prophylactic vaccines. Immense resources were allocated to investigate the biology of SARS-CoV-2 and discover emergency therapeutics to alleviate viral pneumonia, cytokine storm syndromes, thromboembolism, and neurological complications.

Similar to other RNA viruses, SARS-CoV-2 is continuously mutating which challenges initial diagnostic and therapeutic efforts. While the next generation sequencing is indispensable for tracking evolution of SARS-CoV-2 strains across countries and regions, orthogonal -omics approaches, such as mass-spectrometry-based proteomics and metabolomics, offer powerful tools to investigate the functional and clinical impact of the emerging mutations. In this perspective review, we will discuss mass spectrometry-based proteomics as an analytical approach for identification and quantification of SARS-CoV-2 proteins, emerging mutant proteins, homologous proteins of related alpha- and betacoronaviruses, and anti-viral immunoglobulins. Differential quantification of the nearly identical and low-abundance viral proteins is a recognized analytical challenge, and mass spectrometry may emerge as a viable tool to facilitate basic research, environmental monitoring, and clinical diagnostics of SARS-CoV-2.

## Main text

### Human and zoonotic coronaviruses

*Orthocoronavirinae* is a subfamily of 45 single-stranded RNA viruses which infect mammals and birds [1]. Zoonotic alpha- and betacoronaviruses are found in bats, rodents, and rabbits, with bats being the major hosts [2]. The presence of multiple coronaviruses within the related genera of mammals drives the emergence of novel coronavirus strains by shifting between the host species [2]. Extensive global deforestation has been promoting close interactions and cohabitation of species previously residing within the discrete ecological niches. Examples include emerging interactions of horseshoe bats and palm civets (intermediate hosts of SARS-CoV-1), or horseshoe bats and dromedary camels (intermediate hosts of MERS-CoV) [3]. Early evidences indicated that SARS-CoV-2 gained its ability to infect humans through an intermediate mammalian host, such as a pangolin [4]. On a global scale, however, our societal organization, interactions with the wildlife, and economic activities were the actual triggers for MERS and SARS epidemics and pandemics [5].

Alpha- and betacoronavirus genera of *Orthocoronavirinae* subfamily include seven species which can infect humans. Two alphacoronaviruses (HCoV-229E and HCoV-NL63) and two betacoronaviruses (HCoV-OC43 and HCoV-HKU1) are the most common species of the human coronaviruses (HCoVs). These species are associated with “common cold” upper respiratory infections and mild symptoms, and rarely with lower respiratory infections in the elderly or immunocompromised patients [6]. Infections with the remaining three species of betacoronaviruses (SARS-CoV-1, SARS-CoV-2 and MERS-CoV) result in severe clinical manifestations including intense respiratory distress syndromes, systemic hyperinflammation, pulmonary complications, thrombosis, encephalitis, diarrhea, lymphopenia, and multiorgan failure [7], with the case fatality rates of 10%, 6.6%, and 35%, respectively [8, 9]. While several SARS-CoV-2 vaccines have recently been approved [10], no therapies or vaccines against the remaining coronaviruses are available [11].

### Overview of SARS-CoV-2 genome and proteome

**Fig. 1:**
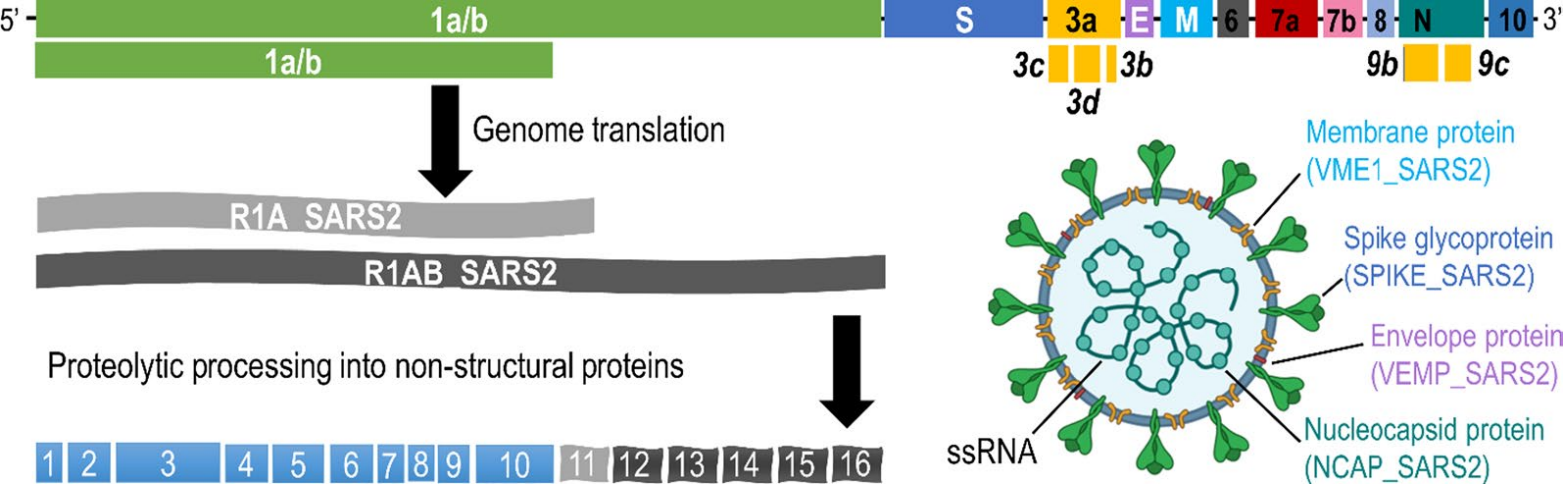
Genome and proteome organization of SARS-CoV-2. Positive-sense single-stranded RNA genome of SARS-CoV-2 (29,903 nucleotides) is composed of two non-structural open reading frames (replicase polyprotein 1a, R1A_SARS2, and replicase polyprotein 1ab, R1AB_SARS2), four structural (spike glycoprotein, SPIKE_SARS2; envelope small membrane protein, VEMP_SARS2; membrane protein, VME1_SARS2; and nucleoprotein, NCAP_SARS2), six accessory non-overlapping open reading frames (ORF3a protein, AP3A_SARS2; ORF6 protein, NS6_SARS2; ORF7a protein, NS7A_SARS2; ORF7b protein, NS7B_SARS2; ORF8 protein, NS8_SARS2; and ORF10 protein, A0A663DJA2_SARS2), and five accessory overlapping open reading frames (ORF3b protein, ORF3B_SARS2; ORF3c protein, ORF3C_SARS2; ORF3d protein, ORF3D_SARS2; ORF9b protein, ORF9B_SARS2; and ORF9c protein, ORF9C_SARS2). Due to a ribosomal frameshift, ORF1ab gene is translated into two replicase polyproteins. Viral proteases PLpro1/PLpro2 (non-structural protein 3; nsp3) and 3CLpro (3C-like proteinase; nsp5) are auto-cleaved from polyproteins and complete the cleavage of the remaining 14 non-structural proteins

Structurally, coronaviruses are the spherical enveloped virions of ~ 100 nm diameter (Fig. 1). The viral genetic material, a positive-sense single-stranded RNA, is associated with ~ 1,000 copies of a nucleoprotein (NCAP_SARS2), and is additionally protected by an envelope small membrane protein (VEMP_SARS2) [12]. Spike glycoprotein (SPIKE_SARS2) homotrimers embedded into the lipid bilayer (~ 100 copies per virion) are essential for the cell invasion and appear as a characteristic crown (from Latin “corona”) under electron microscopy. Coronaviruses have the largest known genomes for RNA viruses (26–32 kilobases) [13]. RNA genome of coronaviruses mutates at a relatively low rate of 10^−4^ nucleotides per site per year [14]. Phylogenetic and mutational analysis of SARS-CoV-2 sequences obtained from 1086 patients revealed its very recent origin from a single predecessor [14].

The entry of coronavirus into host cells is orchestrated by the homotrimer spike glycoprotein. The S1 subunit of spike glycoprotein binds to the cellular receptor ACE2_HUMAN of the host cell [11, 15], and the sequence between S1 and S2 subunits is post-translationally cleaved by two human proteases, furin and TMPRSS2. Such cleavage generates a fusogenic subunit S2 and triggers the virion-cell membrane fusion [11, 16]. It should be noted that TMPRSS2 protease inhibitors efficiently block SARS-CoV-2 cell entry and infection [17]. Recent studies revealed that the structure of the receptor binding domain of SARS-CoV-1 and SARS-CoV-2 were very similar, with the difference of only five amino acids. The S2 subunit was found to be the most conserved region with 93% sequence identity, while S1 subunit revealed only ~ 70% sequence identity to the bat-derived viruses [14].

### Proteomic studies on SARS-CoV-2

#### Identification of SARS-CoV-2 proteins by mass spectrometry

Several studies identified and quantified SARS-CoV-2 proteome by mass spectrometry, in order to reveal expression of the open reading frames, elucidate the viral host response, and identify potential drug targets for therapeutic interventions [18–20]. Using a human colon cancer cell line Caco-2 infected with SARS-CoV-2, Bojkova et al*.* identified and quantified temporal expression of nine SARS-CoV-2 proteins: replicase polyprotein 1ab (R1AB_SARS2), spike glycoprotein (SPIKE_SARS2), ORF3a protein (AP3A_SARS2), membrane protein (VME1_SARS2), ORF6 protein (NS6_SARS2), ORF7a protein (NS7A_SARS2), ORF8 protein (NS8_SARS2), nucleoprotein (NCAP_SARS2), and ORF9b protein (ORF9B_SARS2). The proteome of Caco-2 cells revealed considerable changes after 24 h past SARS-CoV-2 infection, with the distinct impact on splicing, translation, and carbon and nucleic acid metabolism [19]. Gordon et al*.* employed 26 recombinant viral proteins and affinity purification-mass spectrometry to identify 332 high confidence protein–protein interactions in Vero E6 cells [18]. Results included identification of interactions of pp1ab (nsp1) protein with the DNA replication protein PRIM1, formation of a complex between ORF3a protein (AP3A_SARS2) and vesicle trafficking protein VSP11, and binding of ORF9b protein (ORF9B_SARS2) to mitochondrial receptor TOMM70, which could result in suppression of the host cell interferon response. Interaction of ORF6 protein with an interferon-inducible mRNA nuclear export complex NUP98-RAE1 was suggested inhibiting the nuclear transport system. Interestingly, a hypothetical protein ORF10 (TrEMBL UniProt entry A0A663DJA2_SARS2; no evidence of expression in humans [21]) was found associating with Cullin 2 E3 ligase and potentially altering the ubiquitination and protein degradation machinery. Identified protein–protein interactions revealed potential drug targets and small molecule inhibitors of SARS-CoV-2 replication [18].

Recently, Davidson et al*.* investigated SARS-CoV-2 in vitro and in vivo through combination of transcriptomics and proteomics [20]. The study found that SARS-CoV-2 replication in cell culture resulted in a 9-amino acid deletion and removal of a furin-like cleavage site *RRAR* of spike glycoprotein. Since the furin cleavage site was essential for viral entry, its loss during replication in cell culture supported the hypothesis of zoonotic, rather than laboratory, origin of SARS-COV-2 [22]. A recently discovered bat-derived virus RmYN02 comprising insertions in its spike protein similar to those in SPIKE_SARS2 also suggested natural evolution of SARS-CoV-2 [23].

Proteomic studies also identified post-translational modifications of SARS-CoV-2 proteins. Thus, SARS-CoV-2 spike glycoprotein revealed 22 N-linked glycosylation sites [24], similar to 19 sites previously identified for SARS-CoV-1 spike glycoprotein [11, 25]. Since glycosylation may facilitate escape from the host immune system, the diversity of spike glycoprotein glycosylation sites could explain the higher mortality rate of SARS-CoV-2 over SARS-CoV-1 [11]. In addition, 49 phosphorylation sites were identified in SARS-CoV-2 proteins (including nucleoprotein, nsp9, and membrane proteins), and were suggested modulating the host cellular kinase activity and pathways regulating viral replication [26]. It should be noted that one of the first proteomic studies on SARS-related proteins was conducted in 2003 and revealed NCAP_SARS protein as a major immunogen [25]. Interestingly, a caspase cleavage motif (responsible for the virus proteolysis and elimination by the host cells caspases) was present in all previous coronavirus nucleoproteins [27], but not in SARS-COV-1 [25]. Dysregulation of PI3K/AKT pathway was identified in both SARS-CoV-2 and SARS-CoV-1 [28].

#### Quantification of SARS-CoV-2 proteins by targeted mass spectrometry

Quantitative proteomic studies on SARS-CoV-2 proteins revealed that nucleoprotein (NCAP_SARS2) was the most abundant protein contributing up to 90% of all the total proteome [29]. Limit of detection of NCAP_SARS2 parallel reaction monitoring (PRM) assay was in the attomole range (~ 0.9 pg) corresponding to ~ 10,000 SARS-CoV-2 particles. Among all SARS-CoV-2-derived tryptic peptides, GFYAEGSR and ADETQALPQR of nucleoprotein and EITVATSR of membrane protein emerged as peptides with the highest intensities and responsiveness for quantification by mass spectrometry [29]. Likewise, PRM assay of Vero E6 cell supernatant quantified 57 endogenous tryptic peptides corresponding to five viral proteins (SPIKE_SARS2, NCAP_SARS2, VME1_SARS2, ORF9B_SARS2, and NS8_SARS2). Since sequences of some tryptic peptides were identical to those of common cold coronaviruses (229E, HKU1, NL63 and OC43), only 23 peptides were selected for an optimized PRM assay. Some high-intensity peptides (VAGDSGFAAYSR of VME1_SARS2, and LQSLQTYVTQQLIR, FQTLLALHR, and HTPINLVR of SPIKE_SARS2) were well-conserved among human coronaviruses [30]. Since several independent studies revealed identical high-intensity and unique tryptic peptides of NCAP_SARS2 (AYNVTQAFGR, GFYAEGSR, ADETQALPQR) and VME1_SARS2 (EITVATSR, VAGDSGFAAYSR) proteins [29–31], these peptides could be considered as proteotypic peptides for SARS-CoV-2 quantification in biological, clinical, or environmental samples.

### Phenotypic changes of SARS-CoV-2

#### Identification of the emerging mutations

SARS-CoV-2 is continuously mutating, as compared to the original strain isolated in Wuhan, China in January 2020 (GenBank accession MN988668). Early phylogenetic analysis of 160 SARS-CoV-2 genomes revealed three major variants named A, B, and C, with A being the ancestral type originated from the bat coronavirus from Wuhan (GenBank accession MG772933). The B type was derived from A type through the nonsynonymous mutation L84S of ORF8 protein (NS8_SARS2) and adapted to populations outside of East Asia. Both A and C types were found in Europe and the United States, and the C type (harboring G250V mutation in AP3A_SARS2) was the major variant spreading across Europe [32]. Numerous SARS-CoV-2 variants have emerged since then and were rapidly spreading worldwide. The most concerning variants included 20B/501Y.V1 (United Kingdom), 20C/501Y.V2 (South Africa), and 20J/501Y.V3 (Brazil) [33]. These novel variants were harboring missense mutations within S1 domain and receptor-binding domain (RBD) of SPIKE_SARS2 protein, and facilitated stronger binding to the ACE2 receptor [34, 35]. The COVID-CG database is now continuously tracking the real-time evolution and spread of the mutated strains of SARS-CoV-2 across different regions [36]. More than 770,000 SARS-CoV-2 genomes were already summarized, and the most frequent mutations were identified.

#### Perspectives of mass spectrometry to measure SARS-CoV-2 mutations

**Fig. 2:**
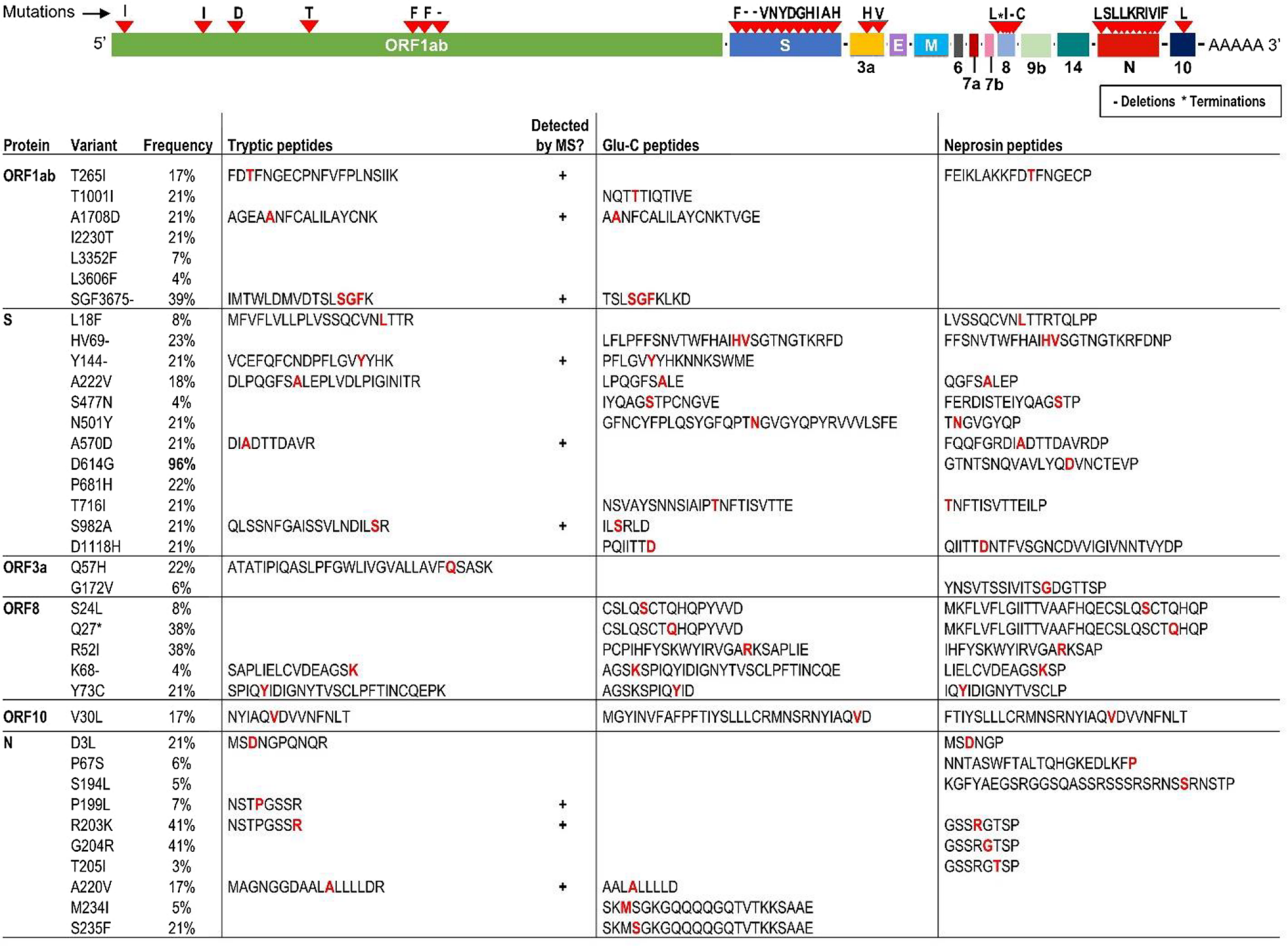
Most frequent nonsynonymous mutations of SARS-CoV-2 proteins and their corresponding trypsin-, Glu-C-, and neprosin-derived peptides. For the 37 most frequent nonsynonymous mutations, deletions (−) and terminations (*) (global frequency > 3%; [36]), 16 reside within the relatively short tryptic peptides (6–31 amino acids). Some of these tryptic peptides were experimentally detected by mass spectrometry [20, 29]. Additional 12 and 14 mutations could be independently detected within Glu-C- and neprosin-derived peptides, thus enabling quantification of ~ 89% of the most frequent SARS-CoV-2 mutations. The most frequent mutation D614G (~ 94%), as well as emerging variants of concern 20B/501Y.V1 (United Kingdom; S N501Y, S A570D, S HV69-, S D614G, ORF8 Q27*, N R203K, N G204R) and 20C/501Y.V2 (South Africa; S N501Y, S D614G) could be detected within the relatively short neprosin-derived peptides [38]

Since standard bottom-up proteomic approaches rely on measurements of relatively short tryptic peptides (which may not harbor nonsynonymous mutations of interest), proteomic approaches utilizing non-tryptic proteases present viable alternatives. Examples include Lys-C (cleaves exclusively after K), Arg-C (cleaves after R), Asp-N (cleaves before D), neprosin (cleaves after P), Glu-C (cleaves after E, or E and D depending on conditions), and others [37–39]. Analysis of the COVID-CG database revealed that the most frequent nonsynonymous mutations within the trypsin-, Glu-C or neprosin-derived peptides could be measurable by liquid chromatography–mass spectrometry. Figure 2 presents the most common nonsynonymous mutations and their frequencies calculated based on ~ 770,000 SARS-CoV-2 genomes [36]. D614G (SPIKE_SARS2) emerged as the dominant nonsynonymous mutation with a frequency > 95% (Fig. 2). For the 37 most frequent nonsynonymous mutations, deletions, and terminations (global frequency > 3%), 16 resided within 6–31 aa tryptic peptides potentially measurable by standard bottom-up proteomic assays (Fig. 2). Additional 12 and 14 mutations could be independently covered with Glu-C- and neprosin-derived peptides, thus providing detection of ~ 89% of the most frequent mutations. While the most frequent mutation D614G could not be detected within short trypsin- or Glu-C-derived peptides (Fig. 2), SPIKE_SARS2 proteolysis by neprosin could enable D614G identification within a 21 aa peptide GTNTSNQVAVLYQDVNCTEVP. Likewise, relatively short neprosin-derived peptides could facilitate measurements of the emerging variants of concern 20B/501Y.V1 (United Kingdom), 20C/501Y.V2 (South Africa), and 20J/501Y.V3 (Brazil) [40–42]. Continuous monitoring of the emerging mutations and their impact on structure and immunogenicity of viral proteins will support studies on SARS-CoV-2 re-infections and future seasonal infections.

#### Functional impact of mutations

Even though viral epidemics are often weakened by emerging mutations interfering with the function of viral proteins [43], there is a possibility that certain mutations may increase viral fitness and drive evolution of more infectious or more deadly strains. The latter could have been a case behind infamous 1918 H1N1 influenza A pandemic which claimed as many as 50 million lives [44].

Accumulation of mutations in the SARS-CoV-2 genome may result in phenotypical changes and functional impact on the cell attachment, membrane fusion, viral replication, and exocytosis. Recent studies identified not only mutations favoring infections, such as a furin cleavage site *RRAR* of SARS-CoV-2 spike glycoprotein [45], but also mutations leading to mild infections, such as ∆382 of NS8_SARS2 protein [46]. Recent X-ray crystallography study revealed that SPIKE_SARS2 residues D936 (as compared to E918 of SPIKE_SARS) and S943 (as compared to T925) facilitated formation of salt bridges and hydrogen bonds, resulting in higher receptor binding affinities and higher infectivity [47]. Likewise, M58R mutation impaired the binding of NS6_SARS2 protein to the Nup98-Rae1 complex and abolished interferon antagonist function of the wildtype NS6_SARS2 protein [48]. Some mutations of AP3A_SARS2 provided alternative B-cell epitopes within the polyproline regions and promoted immune evasion [49]; investigation of AP3A_SARS2 mutants also confirmed their potential to induce apoptosis [50]. Interestingly, preliminary studies suggested that the most frequent mutation D614G was neutral to the function of SPIKE_SARS2; additional studies, however, will be required to assess D614G impact on protein conformation after its interaction with ACE2 [51]. D614G mutation retained within the emerging variants of concern 20B/501Y.V1 (United Kingdom), 20C/501Y.V2 (South Africa), and 20J/501Y.V3 (Brazil), with all three variants having an additional mutation N501Y and resulting in a higher affinity of spike glycoprotein for human ACE2 [33, 40]. E484K mutation shared between the South African and Brazilian variants raised serious concerns due to its increased resistance to antibody neutralization, potentially making some vaccines less effective [41, 52, 53]. Occurrence of both E484K and N501Y mutations in novel variants suggested active and rapid evolution of SARS-CoV-2 [54, 55].

Investigation of SARS-CoV-2 mutations revealed that non-structural proteins 3CLpro (nsp5), PLpro1/PLpro2 (nsp3), and RdRp (nsp12) were highly conserved between SARS-CoV-1 and SARS-CoV-2 and could emerge as promising therapeutic targets [56]. Host translation inhibitor nsp1, a well characterized protein with a conserved biological function [57] among SARS-CoVs and bat CoVs, was found promoting degradation of host mRNA and inhibiting the host innate immune system, while mutations of nsp1 resulted in high levels of interferon beta [58, 59]. Function and enzymatic activities of other non-structural proteins were predicted to be conserved and essential for viral replication.

It should be emphasized that proteogenomic approaches combining mass spectrometry and RNA sequencing data facilitate detailed characterization of novel nonsynonymous mutations and their impact on protein expression, post-translational modifications, or phenotypic variations [60–63]. For example, mass spectrometry was previously used to annotate unknown regions of the bovine herpes virus BoHV-1 genome and identify a novel protein-coding gene ORF-A [64]. Another mass spectrometry study utilizing de novo peptide sequencing detected three amino acid substitutions within nucleocapsid protein of influenza A virus [65]. It is apparent that such knowledge on structure and function of viral proteins could not be obtained by RT-PCR or RNA sequencing alone.

### Perspectives of clinical proteomics and mass spectrometry for COVID-19 diagnostics

#### Serology diagnostics and immunogenicity of mutant strains

**Fig. 3:**
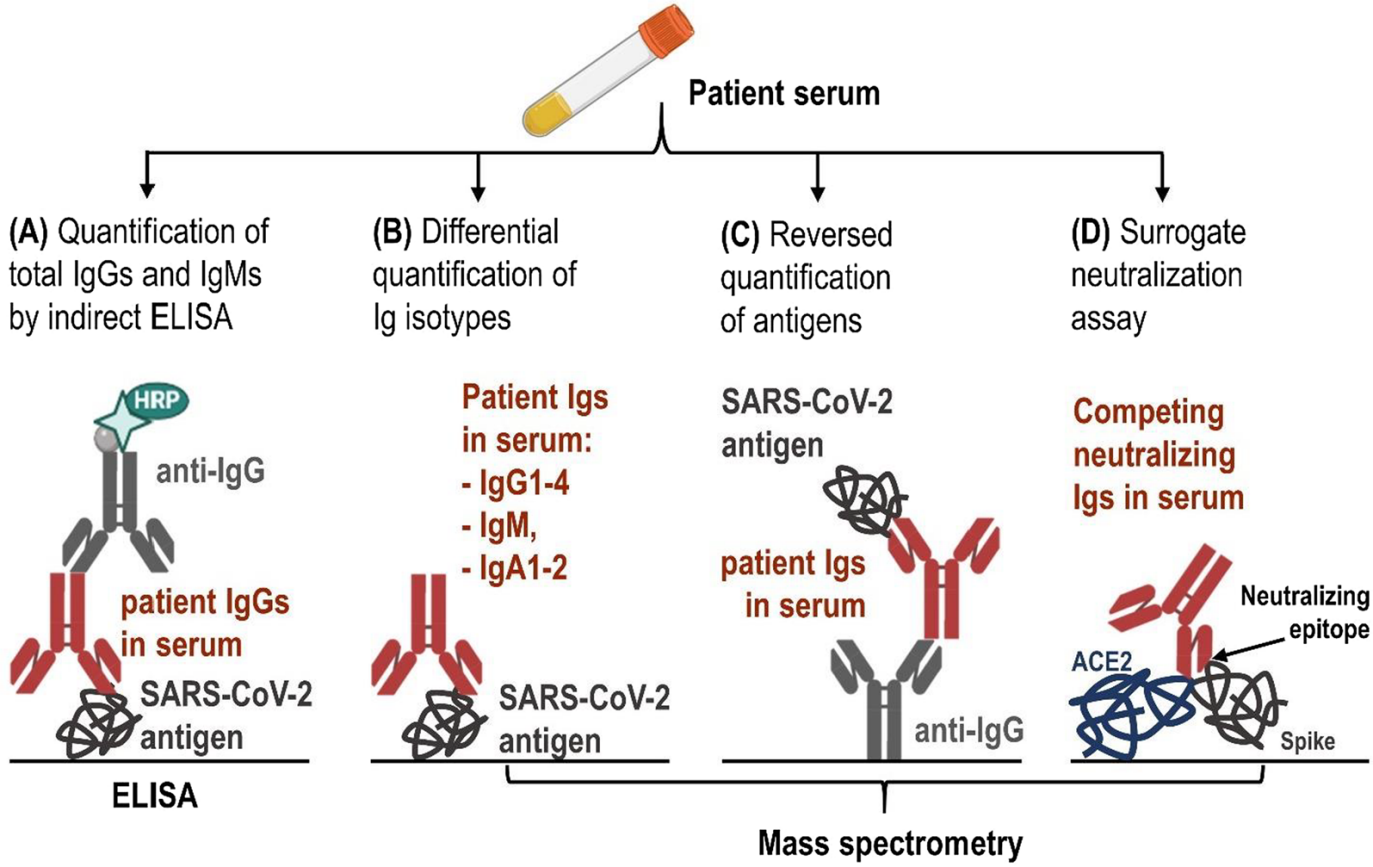
Approaches for quantification of anti-SARS-CoV-2 immunoglobulins. **A** The setup of common serology tests using indirect ELISA allows for quantification of total IgG immunoglobulins, with no differentiation of distinct subclasses. Alternative assays by IP-SRM **B**–**D** facilitate comprehensive investigation of serological response. **B** IP-SRM assays allow for the direct, multiplex and differential quantification of immunoglobulin isotypes (IgG, IgM, IgA) and subclasses (IgG1-4, and IgA1-2) binding to protein antigens or linear epitopes coated onto microwell plates. “Absolute” quantification (pmol/mL or ng/mL) allows for the inter-laboratory standardization of serology assays using stable isotope-labeled peptide internal standards, and for investigation of cross-reactivity of the indirect ELISA. **C** Indirect IP-SRM assays provide additional tools for investigation of cross-reactivity of serology tests. **D** Surrogate neutralization assays by IP-SRM allow for quantification of immunoglobulins disrupting ACE2-spike glycoprotein interactions, and investigation of the impact of mutations emerging within the neutralizing linear epitopes

Detection of specific anti-viral antibodies in blood is indispensable to confirm past infections, identify past asymptomatic or mild symptomatic cases, and reveal immune status of the recovered patients or vaccinated populations. Laboratory or point-of-care tests detecting anti-SARS-CoV-2 antibodies are now mainly based on simple and affordable indirect immunoassays (Fig. 3A). A number of COVID-19 serology assays were recently reported [66–68].

Recent serological studies suggested that 13 days was an average time for seroconversion for IgG and IgM immunoglobulins, even though some SARS-CoV-2 patients plateaued for IgG and IgM within the first week [69]. Serum and plasma also exhibited strong reactivity for IgG3, IgM, and IgA [68]. Another study focused on anti-NCAP_SARS2 antibodies identified IgM and IgA antibodies within 5 days after symptom onset, with the positive detection rates of 85% and 93%, respectively; IgG antibodies were identified 14 days after symptom onset, with the positive detection rate of 78% [70]. The IgG antibodies in serum and saliva were still detectable in 3 months after symptom onset, while IgM and IgA levels rapidly decreased [71]. A combination of RBD and NCAP_SARS2 antigens was the most powerful and facilitated detection of recent mild cases using IgG (AUC ≥ 0.99), IgA (AUC ≥ 0.92), and IgM (AUC ≥ 0.79) antibodies [72]. Detection of IgG antibodies using a combination of RBD and NCAP_SARS2 antigens was suggested as the most informative assay for serosurveillance of all severe and mild cases, including older mild infections (> 5 months; AUC ≥ 0.99). Tan et al. detected IgG antibodies in convalescent COVID-19 serum samples that specifically recognized S1 domain [73]. Those studies suggested that serum and saliva were suitable clinical samples for detection of COVID-19 seroconversion based on IgG measurements.

Recombinant RBD protein has been reported as an antigen with ~ 98% diagnostic sensitivity and ~ 100% diagnostic specificity for detection of anti-viral IgG and IgM antibodies as early as 9 days after of symptoms onset [74]. RBD also distinguished anti-SARS-CoV-2 antibodies from the “common cold” HCoVs infections. Furthermore, anti-NCAP_SARS2 antibodies were produced earlier than anti-SPIKE_SARS2 antibodies. NCAP_SARS2 was the most sensitive (100%) and specific (100%) antigen for the early detection of SARS-CoV-2 antibodies [75]. These results were in agreement with previous findings for SARS-CoV-1 [76, 77]. It should be emphasized  that not fully validated serology tests may generate false-positive results due to cross-reactivity with antibodies developed against the “common cold” HCoV-229E or HCoV-OC43 infections.

It has previously been established that patients recovered from SARS-CoV-1 developed antibody-mediated immunity for at least two years [78]; the similar outcome could be expected for SARS-CoV-2 infections. For instance, recent studies demonstrated that Moderna mRNA-1273 vaccine induced a high titter of binding and neutralizing antibodies which remained elevated in all participants for at least 3 months after the second vaccination [79]. However, some mutated strains may evade immune response , thus resulting in re-infections, similar to the seasonal influenza infections.

**Fig. 4:**
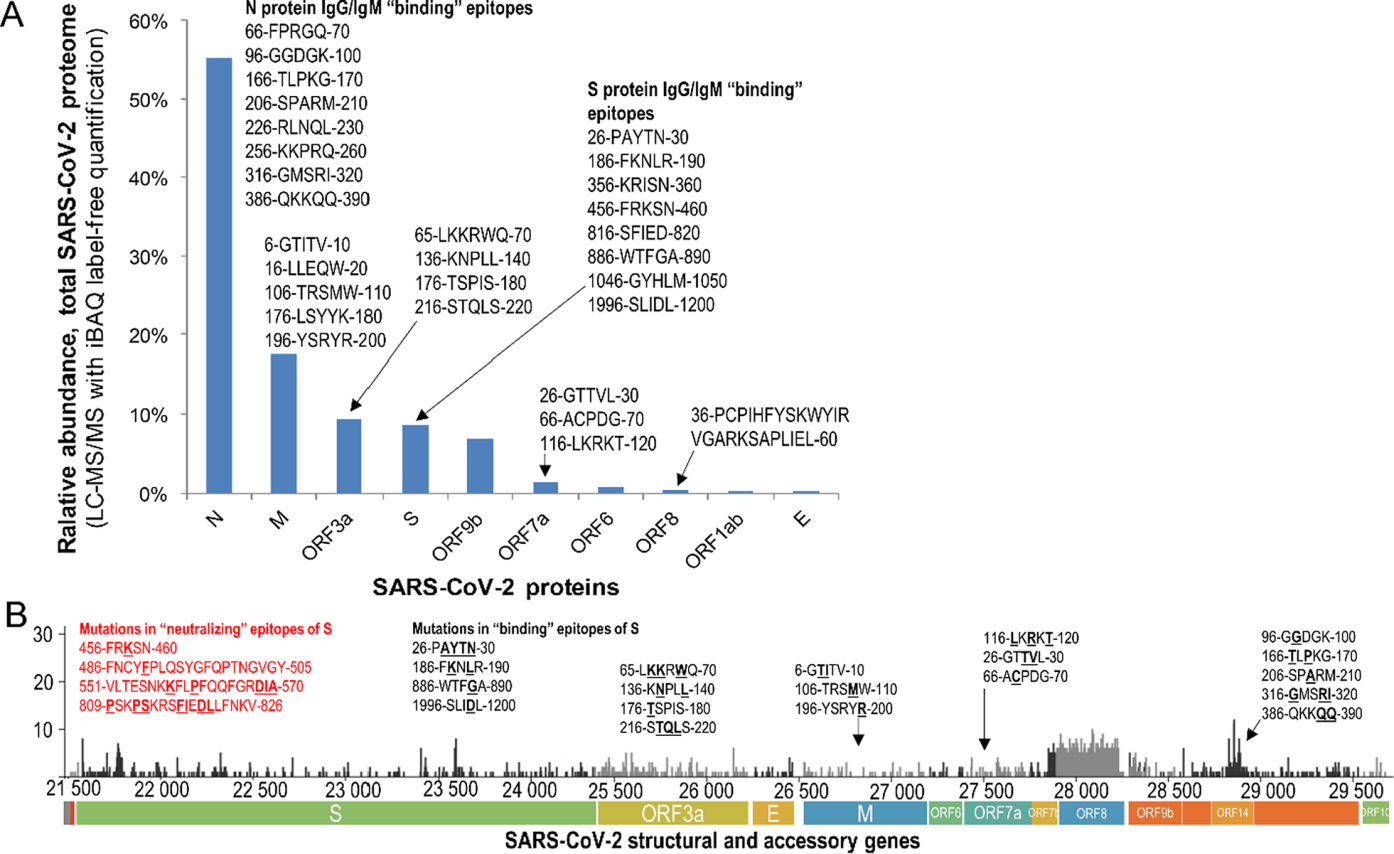
Correlation between the abundance of SARS-CoV-2 proteins and IgG/IgM binding epitopes. **A** Re-analysis of Davidson et al*.* dataset [20] using the label-free intensity-based absolute quantification (iBAQ) approach revealed the relative abundance of SARS-CoV-2 proteins. Interestingly, the relative abundance of SARS-CoV-2 proteins correlated with the number of IgG and IgM binding epitopes identified by Wang et al.[81]. **B** Emerging mutations within the “binding” and confirmed “neutralizing” epitopes of SARS-CoV-2 structural and accessory proteins, according to the GISAID hCoV-19 database [153]. Fourteen mutations within the confirmed “neutralizing” epitopes of S protein (red, underlined, bold) and 35 mutations within the antibody “binding” epitopes (bold, underlined) are presented

Spike glycoprotein was traditionally considered as a key immunogen for vaccine development [80]. Recent studies revealed that some novel mutations were accumulating within the antibody “binding” epitopes (48 novel nonsynonymous mutations) [81] and “neutralizing” epitopes of spike glycoprotein (13 novel mutations) [81, 82] (Fig. 4). Our cross-search of known epitopes and nonsynonymous mutations revealed some concerning mutations of spike glycoprotein (D614G), membrane protein (D3G), and nucleoprotein (G204R) [83, 84]. A neutralizing epitope PSKPSKRSFIEDLLFNKV of spike glycoprotein [81] alone revealed seven mutations. Such mutated epitopes should be evaluated during development of serology tests and vaccines. Interestingly, due to its relatively conserved sequence and high immunogenicity, nucleoprotein was suggested as an alternative target for vaccine development [80].

#### Viral and human proteins as prognostic biomarkers

In the 2000s, some SARS-CoV-1 proteins (R1A_SARS, AP3A_SARS, NS6_SARS and ORF9B_SARS) were suggested as prognostic serum biomarkers to predict the severity of SARS-CoV-1 infections [85–87], cytokine storm syndromes, or pneumonia complications [88]. Validation of SARS-CoV-2 proteins, including accessory proteins, as prognostic biomarkers is still pending due to the lack of high-quality paired antibodies and high-quality immunoassays. Since rational development of protein biomarkers involves numerous phases of discovery, verification and validation, mass spectrometry-based proteomic assays would be particularly useful at the early phases of biomarker development [89, 90].

**Fig. 5:**
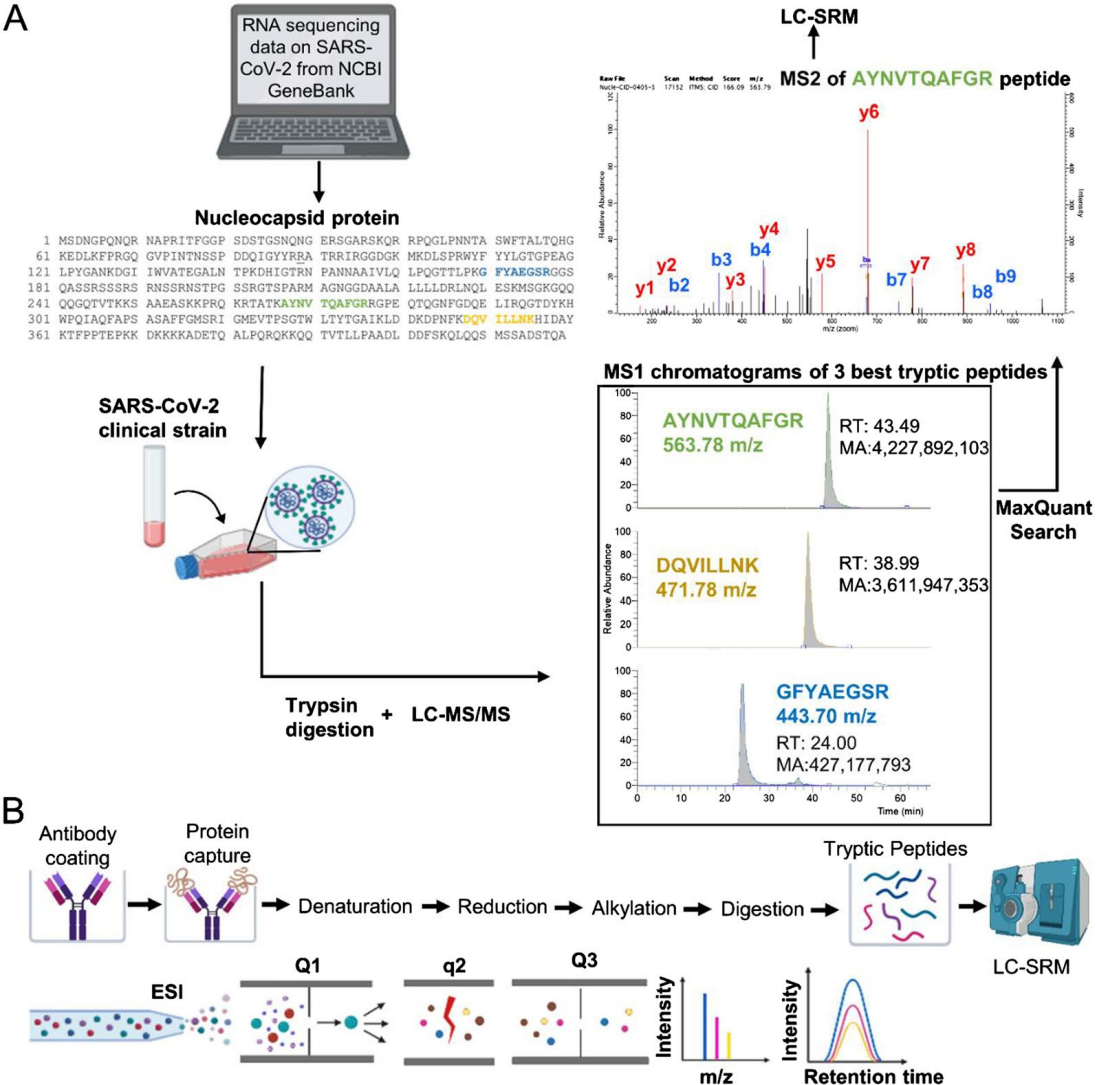
Development of IP-SRM assays. **A** Selection of proteotypic peptides of SARS-COV-2 nucleoprotein based on experimental shotgun mass spectrometry data. Peptide identification by shotgun mass spectrometry provided tryptic peptides with the highest MS1 intensities (our unpublished data), and three peptides AYNVTQAFGR, DQVILLNK and GFYAEGSR were selected. MaxQuant search revealed the most intense y-ion transitions of peptide AYNVTQAFGR. Finally, optimization of the nanoflow liquid chromatography and targeted mass spectrometry parameters generated a quantitative SRM assay. **B** IP-SRM included an antibody-mediated enrichment of NCAP_SARS2 followed by proteomic sample preparation and SRM quantification

While mass spectrometry may not be sensitive enough for the direct measurements of SARS-CoV-2 non-structural and accessory proteins in serum, immunoprecipitation (IP)-targeted proteomic assays (such as selected reaction monitoring, SRM, or parallel reaction monitoring, PRM) present viable alternatives for the preliminary validation of these proteins as prognostic biomarkers in clinical samples (Fig. 5B). Since SRM quantification provides the required analytical selectivity [91, 92], IP-SRM assays may utilize even the low quality polyclonal antibodies, or polyclonal antibodies previously developed against highly homologous SARS-CoV-1 or MERS-CoV proteins.

In addition to SARS-CoV-2 proteins, some circulating human proteins may also emerge as prognostic biomarkers of COVID-19 severity or complications [93, 94]. For example, patients in the severe group revealed significantly higher levels of C-reactive protein and interleukin-6 in serum [95]. Likewise, d-dimers > 1 µg/mL (by-products of the blood clotting and fibrin degradation) facilitated identification of patients with poor prognosis and risk of ischemia and thrombosis [96]. Overall, elevated D-dimer levels suggesting abnormal blood coagulation were common among non-survivor COVID-19 patients [97] and served as biomarkers to predict mortality rates of COVID-19 patients [98, 99].

To design a large-scale proteomic study on prognostic biomarkers, Messner et al*.* developed an ultra-high throughput clinical platform (~ 180 samples/day on a single mass spectrometer) to analyze blood serum and plasma collected from patients with the different WHO severity grades of COVID-19 [100]. As a result, 27 potential prognostic biomarkers (including complement factors, coagulation proteins, inflammation modulators, and pro-inflammatory factors induced by IL-6) were identified. Another study on serum proteome of patients with the severe and mild infections revealed differential expression of 91 proteins, of which 77 proteins were associated with neutrophil degranulation, blood coagulation, and neutrophil mediated immunity, and were proposed as prognostic biomarkers [101].

#### Detection of SARS-CoV-2 in clinical samples by mass spectrometry

While RT-PCR remains the most sensitive and specific assay to diagnose COVID-19 through detection of SARS-CoV-2 mRNA in various clinical samples (pharyngeal swabs, nasal samples, saliva, bronchoalveolar lavage, serum, urine, and feces [102–104]), alternative approaches for quantification of SARS-CoV-2 proteins by mass spectrometry are emerging.

Earlier proteomic studies demonstrated that PRM quantification of SARS-CoV-2 proteins in clinical respiratory specimens was confined to individuals with very high viral loads. Only 11 of 54 RT-PCR-confirmed respiratory specimens (nasopharyngeal swabs and bronchoalveolar lavage) were tested positive for nucleoprotein by PRM assay [31]. Interestingly, potential cross-reactivity of RT-PCR with other coronaviruses was found in two specimens, as revealed by unambiguous identification of peptides shared among common human coronaviruses, while no unique SARS-CoV-2 peptides were detected. Independent mass spectrometry studies detected SARS-CoV-2 proteins in nasopharyngeal swabs and gargle samples, and confirmed that nucleoprotein was the most abundant protein and the best candidate for mass spectrometry detection of SARS-CoV-2 infection [105, 106].

MALDI-TOF has previously been suggested as a technique for unambiguous identification of HCoVs, and for monitoring of emerging pathogens [107]. Nachtigall et al*.* reported MALDI-TOF detection of SARS-COV-2 in nasal swab samples with an accuracy of 94% [108]. A recent study has also utilized analysis of blood serum samples by MALDI-TOF to distinguish COVID-19 patients from negative controls [109]. Relative to RT-PCR analysis of nasal swabs, MALDI-TOF analysis of serum was suggested as a convenient assay due to the direct use of serum after sterilization, low volumes (5 μL), rapid measurements (< 1 min), and lower cross-contamination. Comparison of RT-PCR and MALDI-TOF revealed an acceptable conformity rate (> 80%), and MALDI-TOF was suggested as a surrogate technique for the routine SARS-CoV-2 diagnostics [108]. Analysis of saliva and gargle samples by MALDI-TOF revealed ~ 100% sensitivity to detect SARS-CoV2, and the spike protein fragment S1 was proposed as the marker for SARS-CoV2 screening. SARS-CoV-2 positive samples also revealed elevated levels of IgA immunoglobulins, which could not be measured by RT-PCR assays [110].

#### Differential diagnosis of viral co-infections by mass spectrometry

While RT-PCR is a “gold standard” assay for clinical diagnostics of viral infections and seasonal co-infections [111], proteomics by mass spectrometry may emerge as an alternative approach. Mass spectrometry may find a niche in clinical diagnostics in case of increased diagnostic capacity needs, shortage of RT-PCR reagents, or as an alternative screening tool to complement RT-PCR or immunoassays [112–114].

MALDI-TOF mass spectrometry with its high specificity, rapid turnaround time, automation, and lower costs has recently revolutionized bacterial subtyping [113]. Pending assay development, mass spectrometry could provide differential diagnosis of simultaneous seasonal viral infections. For instance, MALDI-TOF was demonstrated as a promising technique to characterize nonsynonymous mutations of influenza A [65]. In addition, shotgun mass spectrometry allowed for discrimination between two strains of influenza A, H3N2 and H1N1, in the throat swabs [115]. Measurements of signature peptides of neuraminidase of the 2009 H1N1 influenza pandemic by mass spectrometry enabled differentiation of H1N1 from all other strains, including H5N1 [116]. Likewise, measurement of influenza A nucleocapsid protein differentiated between type A H1N1, H3N2, and type B strains [117]. MALDI-TOF proved itself as a tool for the rapid identification of three poliovirus serotypes [118] and differentiation between six known HCoVs (229E, OC43, NL63, HKU1, SARS-CoV, and MERS-CoV) [107], as well as diagnosis of co-infected patients [119].

It should be emphasized that early “common cold” symptoms are not informative to differentiate any of the seven coronavirus infections in human. Furthermore, respiratory tract infections could be associated with the viral and bacterial co-infections in significant number of patients. For instance, some patients were found simultaneously infected with HCoV-OC43 and influenza type A [120], or SARS-CoV-2 and influenza A [121]. Co-infections with influenza type A and B, respiratory syncytial virus type A and B, and parainfluenza type 3 were also known [120].

While in this review we focused on mass spectrometry-based proteomics, it should be noted that mass spectrometry is being increasingly utilized for diagnosis of respiratory viruses through the detection of RT-PCR products [122]. Mass spectrometry enables accurate, rapid and high-throughput (1500 samples/day) measurements of PCR products including single nucleotide polymorphisms, thus permitting the distinction between the nearly identical viral strains. PCR or RT-PCR followed by electrospray ionization in the negative ion-mode and TOF mass spectrometry is emerging as a technology for identification of nearly all known human pathogens directly from clinical samples [123]. Examples include HCoVs differentiation from influenza and respiratory viruses [124, 125]. In addition, RT-PCR/MALDI-TOF assays reveal high agreement with the conventional real-time RT-PCR and equivalent diagnostic sensitivity (97%) and specificity (100%), albeit four times higher limit of detection in saliva (1,563 versus 391 copies/mL) [126].

Detection of high-abundance coronavirus proteins, such as nucleoprotein, may facilitate rapid differentiation of the common strains, diagnosis of coronavirus co-infections, and detection of the emerging strains. Table 1 presents two most intense (our unpublished data), relatively conserved, but unique tryptic peptides of NCAP_SARS2 protein. Quantification of these two variant peptides may facilitate unambiguous differentiation of present and future coronavirus infections and co-infections [116].

**Table 1:** Tryptic peptides suitable for the differential subtyping of seven human coronaviruses

Species	Uniprot name	Uniprot ID	Tryptic peptide	MS1 intensity	Hydrophobicity (SSRCalc)	m/z (+ 2)
SARS-CoV-2	NCAP_SARS2	P0DTC9	AYNVTQAFGR	4 × 10^9^	19.6	563.8
SARS-CoV-1	NCAP_CVHSA	P59595	QYNVTQAFGR		20.0	592.3
MERS-CoV	NCAP_CVEMC	K9N4V7	SFNMVQAFGLR		36.0	635.3
HCoV-OC43	NCAP_CVHOC	P33469	QCTVQQCFGK		14.4	628.3
HCoV-HKU1	NCAP_CVHN1	Q5MQC6	HCNVQQCFGK		11.7	639.3
HCoV-NL63	NCAP_CVHNL	Q6Q1R8	EENVIQCFGPR		24.3	674.8
HCoV-229E	NCAP_CVH22	P15130	QPNDDVTSNVTQCFGPR		22.9	967.9
SARS-CoV-2	NCAP_SARS2	P0DTC9	GPEQTQGNFGDQELIR	1 × 10^9^	24.4	894.9
SARS-CoV-1	NCAP_CVHSA	P59595	GPEQTQGNFGDQDLIR		23.8	887.9
MERS-CoV	NCAP_CVEMC	K9N4V7	GPGDLQGNFGDLQLNK		30.6	836.9
HCoV-OC43	NCAP_CVHOC	P33469	GPNQNFGGGEMLK		22.5	674.8
HCoV-HKU1	NCAP_CVHN1	Q5MQC6	GPSQNFGNAEMLK		24.4	696.8
HCoV-NL63	NCAP_CVHNL	Q6Q1R8	DFNHNMGDSDLVQNGVDAK		24.9	1038.5
HCoV-229E	NCAP_CVH22	P15130	DLDHNFGSAGVVANGVK		26.1	850.4

Presented peptides include the most intense (our unpublished data) and highly conserved, albeit unique, tryptic peptides of the human coronavirus nucleoproteins. Relative hydrophobicity of peptides was calculated with the Sequence Specific Retention Calculator (SSRCalc; http://hs2.proteome.ca/SSRCalc/SSRCalcX.html)

#### Perspectives of immunoprecipitation-mass spectrometry for quantification of viral proteins and serology diagnostics

Immunoprecipitation-mass spectrometry assays combine advantages of two worlds: immunoassays with their high analytical sensitivity, and mass spectrometry with its near-absolute analytical specificity [127, 128]. Targeted proteomic assays were demonstrated as robust tools to quantify proteins in human cell lines [129, 130], primary cells [131–133], tissues [134], various biological fluids [135–139], and blood serum [140]. Steps of IP-SRM development for nucleoprotein (NCAP_SARS2) are presented at Fig. 5A (our unpublished data). Immunoprecipitation provides 100- to 1000-fold gain in sensitivity and enables quantification of low-abundance proteins (LOD ~ 0.1 ng/mL) in clinical samples. Internal standards based on synthetic heavy isotope-labeled peptides with trypsin-cleavable tags facilitate “absolute quantification” (pmol/mL or ng/mL) of proteins in clinical samples [140]. All steps of immunoprecipitation and proteomic sample preparation could be implemented on multiwell microtiter plates and provide high reproducibility and sufficient throughput (~ 20 samples per day). Various types of hybrid mass spectrometers (triple quadrupoles, quadrupole-ion traps, quadrupole-Orbitraps, and etc.) support targeted quantification of unique tryptic peptides. When combined with immunoprecipitation and liquid chromatography, IP-LC-SRM assays provide four stages of analyte enrichment and separation, and emerge as highly selective assays for protein quantification [141].

IP-MS and IP-SRM has previously been used to analyze Ebola, influenza, and hepatitis C proteins [142–144]. IP-MS confirmed interaction of ICP27 protein of herpes simplex virus type 1 (HSV-1) with the cellular translation initiation factors [145], identified proteins interacting with ICP28 protein [146], and confirmed association of a single-stranded DNA-binding protein ICP8 with numerous host proteins involved in the cellular DNA replication, DNA damage repair, chromatin remodeling, and mRNA splicing [146]. Likewise, novel interactions of influenza A ribonucleoprotein with the nuclear transport protein importin-β3 and the regulatory protein PARP-1 were discovered by IP-MS [147]. Sensitivity of IP-MS assay was sufficient to quantify influenza hemagglutinins in purified virus and commercial vaccines [148]. IP-SRM has also been suggested as a rapid response assay for future influenza pandemics [148, 149]. With respect to IP-MS for human coronaviruse studies, ACE2 and dipeptidyl peptidase 4 (DPP4) were identified as receptors for SARS-CoV-1 and MERS-CoV, respectively [150, 151].

IP-MS and IP-SRM assays could find applications as alternative tools for highly specific and sensitive quantification of SARS-CoV-2 proteins, and for independent evaluation of serology tests (Fig. 3). Thus, the complete antibody‐mediated immune response could be measured with a multiplex IP-SRM assay targeting all human antibody isotypes and subclasses (IgG1-4, IgM, IgA1-2, IgD, and IgE). Similar to validation of the conserved and immunogenic antigens of parasitic microorganisms [152], evaluation of the numerous wildtype and mutated linear epitopes by IP-MS might also reveal the most conservative antigens and promising epitopes for development of monoclonal antibody therapies or universal vaccines.

## Conclusions and future perspectives

In this review, we described perspectives of mass spectrometry as an analytical technique to identify and quantify SARS-CoV-2 proteins and enable basic research and clinical studies on COVID-19. While RT-PCR and RNA sequencing are indisputably powerful techniques for detection of SARS-CoV-2 and identification of emerging mutations, proteomics by mass spectrometry may generate novel knowledge which cannot be derived from RNA sequences. Examples include the abundance of viral proteins, post-translational modifications, protein–protein interactions, and the impact of emerging mutations on protein structure and function.

LC-MS, MALDI-TOF and RT-PCR/MS present promising mass spectrometry approaches for COVID-19 diagnostics in clinical laboratories. Among those, MALDI-TOF is a well-established technique available in the large microbiology laboratories for identification of bacterial and fungal infections, and has already been demonstrated for diagnosis of SARS-CoV-2 infections [107–110]. Although the throughput of IP-MS assays (as compared to MALDI-TOF and RT-PCR/MALDI-TOF) is not sufficient for the routine diagnostic testing, IP-MS will find certain applications in biomedical research, including studies on protein–protein interactions, measurement of the low-abundance mutant proteins, assessment of the functional impact of novel mutations, and investigation of cross-reactivity of serology tests.

The COVID-19 pandemic is another reminder that the future global pandemics are inevitable due to the increased interaction between wild animals, livestock, and humans. The threat of future epidemics, however, could be mitigated through the continuous monitoring of emerging mutations of zoonotic and human viruses and predicting the risk of animal-to-human or human-to-human transmission of the emerging strains.

## Data Availability

The datasets used and analysed during the current study are available from the corresponding author on reasonable request.

## Abbreviations

aa: Amino acid
ACE2: Angiotensin converting enzyme 2
ELISA: Enzyme linked immunosorbent assay
MALDI: Matrix-assisted desorption ionization
nsp: Non-structural protein
Ig: Immunoglobulin
IP-MS: Immunoprecipitation-mass spectrometry
PRM: Parallel reaction monitoring
SARS: Sever acute respiratory syndrome
SRM: Selected reaction monitoring
